# A Polysaccharide From the Whole Plant of *Plantago asiatica* L. Enhances the Antitumor Activity of Dendritic Cell-Based Immunotherapy Against Breast Cancer

**DOI:** 10.3389/fphar.2021.678865

**Published:** 2021-08-24

**Authors:** Jiafeng Gao, Yi-Nan Zhang, Jingwen Cui, Jiatong Zhang, Yuexiang Ming, Zhihui Hao, Huihao Xu, Nan Cheng, Di Zhang, Yipeng Jin, Degui Lin, Jiahao Lin

**Affiliations:** ^1^ The Clinical Department, College of Veterinary Medicine, China Agricultural University, Beijing, China; ^2^ Institute of Biomaterials and Biomedical Engineering, University of Toronto, Toronto, Toronto, Canada; ^3^ Center of Research and Innovation of Chinese Traditional Veterinary Medicine, China Agricultural University, Beijing, China

**Keywords:** *Plantago asiatica* L, dendritic cells, cytotoxic T cells, breast tumor, immunotherapy

## Abstract

Dendritic cells (DCs) are the most potent professional antigen-presenting cells (APCs) that mediate T-cell immune responses. Breast cancer is one of the most commonly diagnosed diseases and its mortality rate is higher than any other cancer in both humans and canines. Plantain polysaccharide (PLP), extracted from the whole plant of *Plantago asiatica* L., could promote the maturation of DCs. In this research, we found that PLP could upregulate the maturation of DCs both *in vitro* and *in vivo*. PLP-activated DCs could stimulate lymphocytes’ proliferation and differentiate naive T cells into cytotoxic T cells. Tumor antigen-specific lymphocyte responses were enhanced by PLP and CIPp canine breast tumor cells lysate-pulsed DCs, and PLP and CIPp-cell-lysate jointly stimulated DCs cocultured with lymphocytes having the great cytotoxicity on CIPp cells. In the 4T1 murine breast tumor model, PLP could control the size of breast tumors and improve immunity by recruiting DCs, macrophages, and CD4^+^ and CD8^+^ T cells in the tumor microenvironment. These results indicated that PLP could achieve immunotherapeutic effects and improve immunity in the breast tumor model.

## Introduction

Dendritic cells (DCs) act as initiators of the initial immune response and play an important part in the regulation of the immune system. DCs recognize, capture, process, and present antigen to naive T cells, which stimulate the activation and proliferation of naive T cells for adaptive immune responses (Batra et al., 2012). DCs play a crucial role in triggering anticancer T-cell–mediated immunity against cancer cells. It has been confirmed that the number of DCs is closely related to cancer cell infiltration, lymph node metastasis, and prognosis (Steinman and Banchereau 2007; Palucka and Banchereau 2012).

Breast cancer is one of the most commonly diagnosed diseases and its mortality rate is higher than any other cancer in both humans and canines (KH et al., 2018). As an emerging anticancer therapeutic approach, immunotherapy is designed to harness the patient’s own immune system, in which DCs present the cancer antigens to activate antigen-specific T-cell responses for fighting against the cancer cells (Klingemann, 2018). However, within the tumor microenvironment (TME), the function of DCs was inhibited that limits the effective anticancer T-cell responses, probably because the tumor tissue could secrete certain substances to inhibit the function of DCs (Sabado et al., 2017; Dong et al., 2018).

**GRAPHICAL ABSTRACT:**
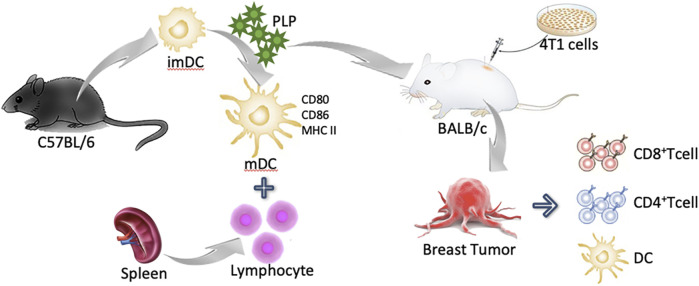


Chinese herbal extracts, especially their polysaccharide components, exhibit validated therapeutic effects and have shown the ability to promote the maturation of DCs. *Rehmannia glutinosa* polysaccharides (Huang et al., 2016), Cordyceps sinensis extracellular polysaccharides (Song et al., 2011), Kudzu root polysaccharides (Kim et al., 2013), Platycodon grandiflorum polysaccharides (Park et al., 2014), and Ganoderma lucidum polysaccharides (Meng et al., 2011) have all been verified to induce the maturation of DCs. Evidence showed that the clinical effects of these polysaccharides are related to the upregulated expression of costimulatory molecules such as CD80, CD86, and MHC II and the activation of tumor-related T-cell response. These studies provide evidence for the use of Chinese herbal extracts in clinically enhancing host immune function.

Our preliminary experiment had screened extractions of ten different Chinese herbal medicines which have been reported to have the ability of immune upregulation. The results revealed that among the ten candidates, including extraction of *Lycium barbarum* L., dried root of *Rehmannia glutinosa* (Gaertn.) DC., *Schisandra chinensis* (Turcz.) Baill., *Artemisia caruifolia* Buch.-Ham. ex Roxb. (syn. *Artemisia* apiacea), *Ganoderma lucidum* (Curtis) P. Karst., *Panax ginseng* C.A.Mey., *Astragalus mongholicus* Bunge, and *Plantago asiatica* L., etc., the total extraction of *Plantago asiatica* L. (PLPt) showed the most excellent ability to activate DCs.

*Plantago asiatica* L., belonging to the Plantaginaceae family, is a perennial plant and is widely distributed in eastern Asia. This plant has not only been used as medicine but has also been approved as raw material for healthy foods with a time-honored history (AB 2000; Yin et al., 2018). Polysaccharides from Plantago leaves have also been reported to induce nitric oxide and TNF-α secretion by activating macrophages (Biringanine et al., 2005). Recent studies mainly focused on the seed of *Plantago asiatica* L. and its purified polysaccharide which could upregulate the expression of maturation markers, decrease DCs’ endocytosis, and increase intracellular interleukin (IL)-12 levels and heterologous stimulus activity, and promote T-cell proliferation (Huang et al., 2009; Huang et al., 2014; Jiang et al., 2018). Compared with the seed, the whole plants of *Plantago asiatica* L. have the advantages of widespread availability, limited cost, and easy processing. Therefore, the polysaccharide extracted from the whole plant of *Plantago asiatica* L. (PLP) has been screened as the main research object in our experiment. We want to assess if the PLP could induce the maturation of DCs and then stimulate the systemic immune reaction.

In this research, we investigated whether PLP could induce the maturation of mouse myelogenous DCs, and the PLP-activated DCs could stimulate lymphocytes’ proliferation and differentiate naive T cells into cytotoxic T cells, which is associated with cellular immune function. Specifically, we evaluated its cytotoxicity on canine breast tumors cell lines and validated its influence on the status of DCs and CTLs against cancer cells. Based on the results, PLP could achieve immunotherapeutic effects and improve immunity in the breast tumor model. In addition, traditional Chinese medicine is expected to play an effective role with DCs in the immunotherapy of breast cancer and to activate the antitumor immune cycle efficiently.

## Materials and Methods

### Animals

6- to 8-week-old female C57BL/6 mice, 4- to 6-week-old female BALB/c mice, and 4- to 6-week-old BALB/c nude mice with cleaning grade were purchased from Beijing Vital River Laboratory Animal Technology Co., Ltd. (Beijing, China, Certificate No. SCXK (Jing) 2016-0008). These animals were taken feeding management with free access to standard chow and water about a week before the experiment to adapt to the environment (24 ± 1°C and 12/12-h light/dark cycle). The mice were fasted overnight except water before the experiment. All animal studies were reviewed and approved by China Agricultural University Laboratory Animal Welfare and Animal Experimental Ethical Committee (Approval ID: CAU 2015121701–1).

### Preparation of PLP

The crude *Plantago asiatica* L. herb polysaccharide was extracted using water extraction and alcohol precipitation method. *Plantago asiatica* L. was a kind of biennial herb, and we bought the standard decoction pieces of *Plantago asiatica* L. from Tongrentang Co., Ltd. (Beijing, China). Firstly, the *Plantago asiatica* L. herb was soaked in absolute ethanol with 8 times the amount of the herb overnight. The absolute ethanol part was then discarded, and the drugs were dried for subsequent use. Secondly, the extraction of dried residues was performed using the reflux extraction method 2 times with 2 h for each time. After the extracts were merged and concentrated, 4 times anhydrous ethanol was added and then the mixture was stored at 4°C overnight. Thirdly, the mixture was centrifuged at 3,500 r/min for 15 min to discard the supernatant, while the precipitates were dried and ground into powder. Then the PLP was obtained and stored at 4°C for further use.

The precipitate was redissolved and deproteinized using the Sevag method (Staub et al., 1965). To purify the PLP, 0.2 g PLP powder was dissolved in 40 ml distilled water. Then 10 ml of chloroform and n-butanol (4:1, v/v) were added into the suspension with fully shaking for 20 min. Centrifugal separation was performed five times at 2,500 r/min for 3 min each time to aspirate the supernatant. After the deproteinization procedure, the protein content of the PLP solution was determined using Micro Bicinchoninic Acid (BCA) protein assay method (Thermo Fisher, United States) according to the manufacturer’s instructions.

Endotoxin was removed and assayed under endotoxin-free experimental conditions by using EtEraser™ HP Endotoxin Removal Kit and Bioendo™ EC Endotoxin Test Kit (Chinese Horseshoe Crab Reagent Manufactory Co., Fujian Province, China) according to the manufacturer’s protocol. In brief, PLP was collected after flowing through the highly efficient endotoxin removal resin prepacked column. After endotoxin removal, 100 μL of PLP, standards, or endotoxin-free water (negative control) was mixed with 100 μL of TAL and the optical density (OD) was measured at 450 nm. The quantity of endotoxin was estimated to be ≤ 0.02 endotoxin unit (EU) per mg of PLP.

### Mouse Bone Marrow–Derived DC Culture

Mouse bone marrow–derived DCs (BMDCs) were prepared with some modifications based on the references (Grauer et al., 2002). Briefly, bone marrow cells were harvested from the femurs and tibias of 6- to 8-week-old female C57BL/6 mice and depleted of erythrocytes by Red Blood Cell Lysis Buffer. Cells were washed and cultured at a concentration of 2 × 10^6^ cells/mL in RPMI-1640 supplemented with 10% FBS, 100 U/mL penicillin, 100 U/mL streptomycin (GIBCO BRL, Germany), and 20 ng/ml recombinant mouse (rm) GM-CSF (PeproTech, United States). The medium was changed every 2 days. On Day 7, non-/loosely adherent cells were harvested and cultured in 24-well plates at 1 × 10^6^ cells/mL. The cells were stimulated for 48 h with LPS (2 μg/ml, Sigma-Aldrich, United States), PLP (50 μg/ml), extraction of *Plantago asiatica* L. (PLPt, 50 μg/ml, purchased from Beijing Dongzhimen Hospital), or acteoside (50 μg/ml, purchased from Baoji GuoKang Biotechnology, China) respectively and then analyzed.

### Cytotoxicity Assay

Cytotoxicity was evaluated using a commercial Cell Counting Kit-8 (Beyotime, China). Briefly, BMDCs were plated in 96-well plates at 1 × 10^4^ cells per well and treated with RPMI-1640 alone (control group) or with different concentrations of PLP (12.5, 25, 50, 100, and 200 μg/ml). After 24 and 48 h, the CCK-8 solution was added to the cell wells and incubated at 37°C for 1 h to follow the instruction. Cell viability was assessed by measuring the optical density (OD) with a microplate reader (ELx808 TM; BioTek Instruments, United States) at 450 nm.

### Maturation Marker Analysis

The effect of PLP on the maturation markers of DCs was examined using flow cytometry. The cultured DCs served as both testing group and control group were harvested on Day 9, and incubated cells were washed by cold PBS containing 2% FBS. Cells were proceeded to be incubated with FITC-conjugated anti-mouse CD11c, PE-conjugated anti-mouse MHC class II (I-A/I-E), PerCP/Cyanine 5.5-conjugated anti-mouse CD80, and APC-conjugated anti-mouse CD86 antibodies (Biolegend, United States) for 30 min at 4°C in the dark. Appropriate isotype-matched monoclonal antibodies served as negative controls. The percentages of CD80, CD86, and MHC class II were detected by a FACS Calibur (BD Pharmingen, United States) and analyzed with FlowJo software (TreeStar, United States).

### Cytokine Assays

Interleukin (IL)-12p70, tumor necrosis factor (TNF)-α, IL-1β, IL-4, and IL-6 from the supernatant of cell culture medium were determined by ELISA kits according to the manufacturer’s instructions (Proteintech, China). The optical density (OD) at 450 nm was measured with a microplate reader (ELx808TM; BioTek Instruments, United States).

### DCs Stimulation with PLP *In Vivo*

• PLP (10 mg/kg) was administered by subcutaneous injection (s.c.), intraperitoneal injection (i.p.), or oral administration (p.o.) to C57BL/6 mice of three groups. Naive mice without PLP injection were used as a negative control, while LPS (2.5 mg/kg) was administered s.c. as a positive control. After 48 h, BMDCs were isolated and cultured until the seventh day to examine the CD80, CD86, and MHC class II expression.

### Mixed Lymphocyte Reaction (MLR)

Mouse spleen lymphocytes were isolated by Mouse spleen lymphocytes Separation Solution kits according to the manufacturer’s instructions (Solarbio, China). Briefly, 6- to 8-week-old female C57BL/6 mice were killed, and the spleens were transferred aseptically to Petri dishes, then cut into small pieces, minced, and filtered to obtain spleen cells suspension. The spleen cell suspension was slowly added into the lymphocyte separation solution in a ratio of 1:1. After centrifugation, the middle milky white cloud portion was taken to obtain spleen lymphocyte suspension. Finally, the density of cell suspension was set at 5.0 × 10^6^ cells/mL in a complete RPMI-1640 medium. Then, spleen lymphocytes were stained with 1.25 μM carboxyfluorescein (CFSE, Biolegend, United States) at 37°C for 20 min in dark. Mature DCs (matured in the presence of PLP or LPS) were pretreated with 50 ng/ml mitomycin C (a selective proliferation inhibitor which abrogates DNA synthesis, Sigma-Aldrich, United States) for 30 min at 37°C. After being thoroughly washed, CFSE-stained lymphocytes were cocultured with mature BMDCs in different groups at a BMDC: lymphocyte ratio of 1:4. After 48 h of coculture, cells were collected and incubated with PE-conjugated anti-mouse CD8a and APC-conjugated anti-mouse CD3 antibodies (Biolegend, United States) for flow cytometry analysis, and culture supernatants were collected for cytokine assays by IFN-γ ELISA kits (Proteintech, China).

### Tumor Immunotherapy of PLP

#### Cell Culture

The CIPp cell line, established from a primary lesion of a female dog diagnosed with canine mammary tubular adenocarcinoma at clinical stage IV, was a generous gift from Professor Nobuo Sasaki (University of Tokyo, Japan). And murine breast cancer cell line 4T1 was purchased from ATCC (American Type Culture Collection, Manassas, VA, United States). The CIPp cells were cultured in Dulbecco’s modified Eagle’s medium (DMEM), supplemented with 10% FBS in a 5% CO_2_ air incubator at 37°С. The 4T1 cells were cultured in RPMI-1640, supplemented with 10% FBS in a 5% CO_2_ air incubator at 37°С.

#### Analysis of Tumor-Specific Immunity

Tumor cell lysates were prepared by five freeze–thaw cycles of CIPp cells (1 × 10^7^ cells/mL in PBS). Cellular debris was removed by centrifugation, and the lysate solution was passed through a 0.22 μm membrane filter and stored at −80°C.

BMDCs were cultured in 24-well plates at 1 × 10^6^ cells/mL were stimulated for 48 h with LPS (2 μg/ml), PLP (50 μg/ml), CIPp-cell-lysate, or CIPp-cell-lysate + PLP. DCs were incubated with tumor lysates at a ratio of 3:1 tumor cell equivalent. The stimulated DCs were collected to coculture with lymphocytes for tumor-specific immunity analysis.

Tumor-specific lymphocyte proliferation was determined by a similar method as MLR. Briefly, spleen lymphocytes were stained with 1.25 μM carboxyfluorescein (CFSE, Biolegend, United States) at 37°C for 20 min in dark. After being thoroughly washed, CFSE-stained lymphocytes were cocultured with BMDCs in different groups (LPS, PLP, CIPp-cell-lysate, and CIPp-cell-lysate + PLP) at a BMDC: lymphocyte ratio of 1:4. After 48 h of coculture, cells were collected for flow cytometry analysis. And the supernatants from the aforementioned groups were collected and centrifuged at 1,000 rpm for 10 min. The finally obtained supernatants were collected and stored at −80°C as conditioned medium.

#### CIPp Inhibitory Effects of PLP and Conditioned Medium

CCK-8 assay was conducted to investigate the effect of PLP and different groups of conditioned medium on the viability of CIPp cells. Cell viability assay was performed by seeding CIPp cells in a 96-well microplate at a density of 1 × 10^4^ cells per well for 24 h before attached. Then cells were divided into different groups including the control group (DMEM) and groups treated with different concentrations of PLP (8, 15, 30, 60, 125, and 250 μg/ml). Cell viability was assessed with a commercial Cell Counting Kit-8 (Beyotime, China) at 24 and 48 h posttreatment according to the manufacturer’s instructions. And different groups of conditioned medium (LPS, PLP, CIPp-cell-lysate, and CIPp-cell-lysate + PLP) were added into CIPp cell culture medium for 48 h. The optical density (OD) was measured with a microplate reader (ELx808TM; BioTek Instruments, United States) at 450 nm. All experiments were performed in quadruplicate.

To confirm the impact of PLP-associated T-cell immunity on tumor inhibition, we administered PLP (10 mg/kg) to CIPp tumor-bearing BALB/c nude mice which reveal congenital defects of T cells. BALB/c nude mice were s.c. inoculated with CIPp cells (1 × 10^5^ cells). When tumor size reached approximately 100–200 mm^3^ in volume, the mice were administered physiological saline and PLP (10 mg/kg/d, s.c.) every 24 h. The tumor volume was calculated using the following formula: mm^3^ = (longest diameter × shortest diameter^2^)/2. Mice were sacrificed at Day 28.

#### Tumor Experiments

4- to 6-week-old female BALB/c mice were used for the breast tumor-bearing model. Subcutaneous tumor models were established by inoculating 5 × 10^5^ 4T1 cells into the right flanks of the BALB/c mice. When tumor size reached approximately 100–200 mm^3^ in volume, the mice were administered physiological saline, PLP (10 mg/kg/d, s.c.) once every 24 h, and TAXOL^®^ (Harbin Pharmaceutical Group Holding Co., Ltd., Heilongjiang, China, 6 mg/kg, i.p.) and TAXOL^®^+PLP once every 4 days. Mice were closely monitored every other day for pain/distress, tumor volume, and body weight. The tumor volume was calculated using the following formula: mm^3^ = (longest diameter × shortest diameter^2^)/2. Mice were sacrificed at Day 22. Tumors were removed and weighed, and tumors, spleens, and inguinal lymph nodes were excised for flow cytometer analysis of immune cell subpopulations. Cells were proceeded to be incubated with FITC anti-mouse CD11c, APC anti-mouse CD3, PE anti-mouse CD8a, PerCP/Cyanine5.5 anti-mouse CD4, FITC anti-mouse IFN-γ, PE/Cyanine7 anti-mouse CD11b, and PE anti-mouse F4/80 antibodies (Biolegend, United States) for 30 min at 4°C in the dark. Cellular supernatant was collected for IFN-γ ELISA analysis.

### Statistical Analysis

Statistical analyses were performed using Prism GraphPad 7 software. Each experiment was repeated at least three times. The quantitative data collected were expressed as mean ± S.D. One-way analysis of variance (ANOVA) with Dunnett’s posttest was used for analysis of multiple groups, considered statistically significant if *p* < 0.05 (*p < 0.05, **p < 0.01, and ***p < 0.001, unless otherwise indicated).

## Results

### Morphological Changes in Dendritic Cells after Treatment and Cytotoxicity Evaluation

**FIGURE 1:**
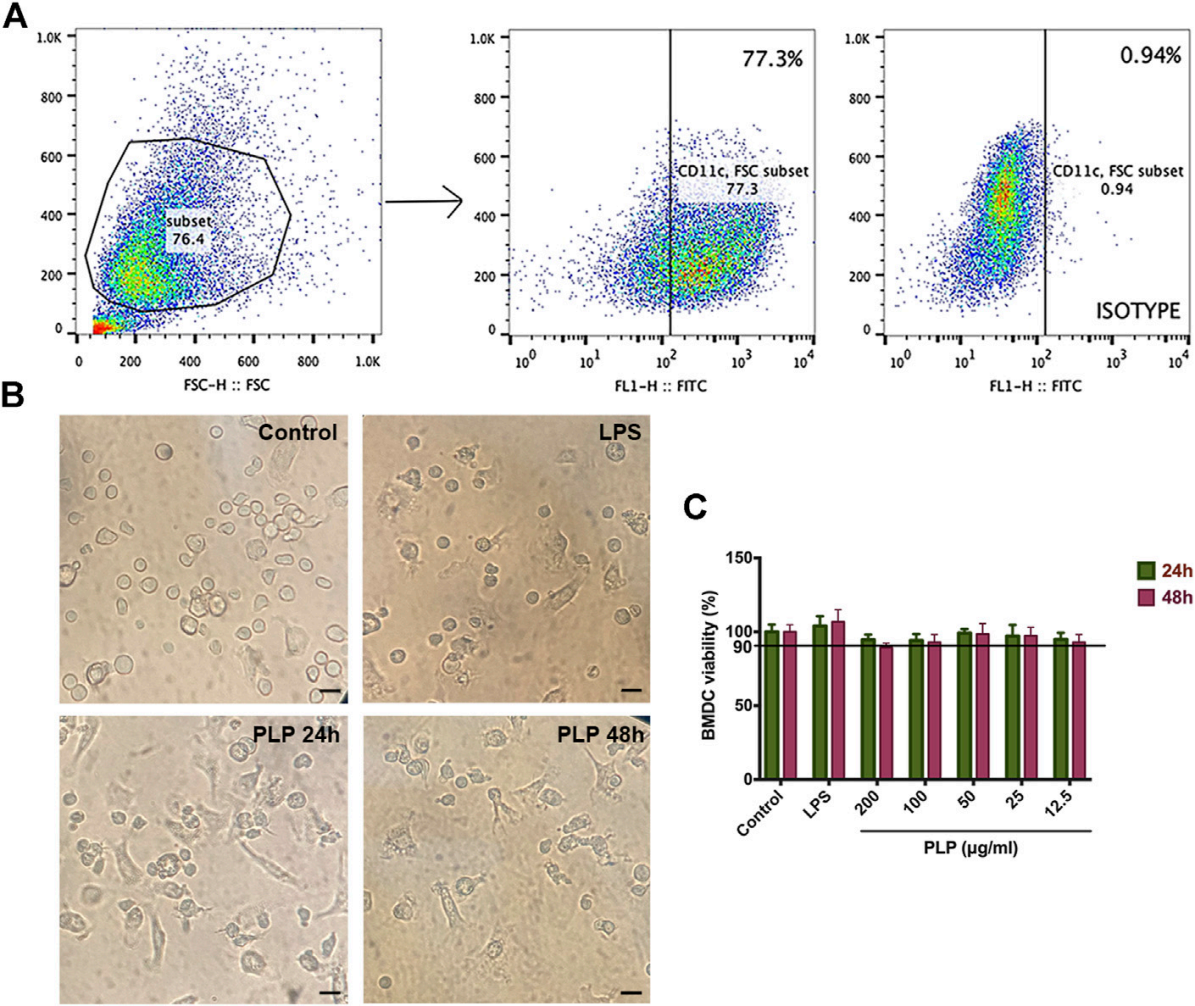
Purity and morphological changes in dendritic cells and cytotoxicity evaluation. **(A)** Subsets of CD11c^+^ cells were gated. **(B)** Representative microscopic views of BMDCs maturation stimulated with PLP (50 μg/ml) and LPS (positive control) for 48 h (×100). Bar = 150 μm. **(C)** Cytotoxic effects of DCs were analyzed using Cell Counting Kit-8 at 12.5, 25, 50, 100, and 200 μg/ml PLP treatment. Data are shown as mean ± SEM (n = 4) (*p < 0.05, **p < 0.01, and ***p < 0.001).

We studied the DC population, defined as CD11c^+^ cells using flow cytometry (Figure 1A). After being cultured for 7 days, the purity of CD11c^+^ cells was detected by flow cytometry with a positive rate of 77.3%, which can be used in the subsequent experiments.

We observed the biological characteristics of BMDCs, and the results are shown in Figure 1B. In the control group, the light microscope showed that the semisuspended DCs had a round shape. In the groups treated with PLP and LPS, morphological characteristics of DCs changed to be enlarged, suspended, and displayed more typical dendritic protrusions. These changes in morphological characteristics suggested that DCs treated with PLP were more liable to convert into mature dendritic cells.

We tested the potential cell toxicity of PLP by incubating the DCs with different concentrations of PLP, LPS, and RPMI-1640, respectively. The DCs’ viability was assessed by CCK-8 assay. No evidence of DCs toxicity *in vitro* could be observed within the concentration range of PLP from 12.5 to 200 μg/ml (Figure 1C).

### PLP Induced DC Maturation *In Vitro*

**FIGURE 2:**
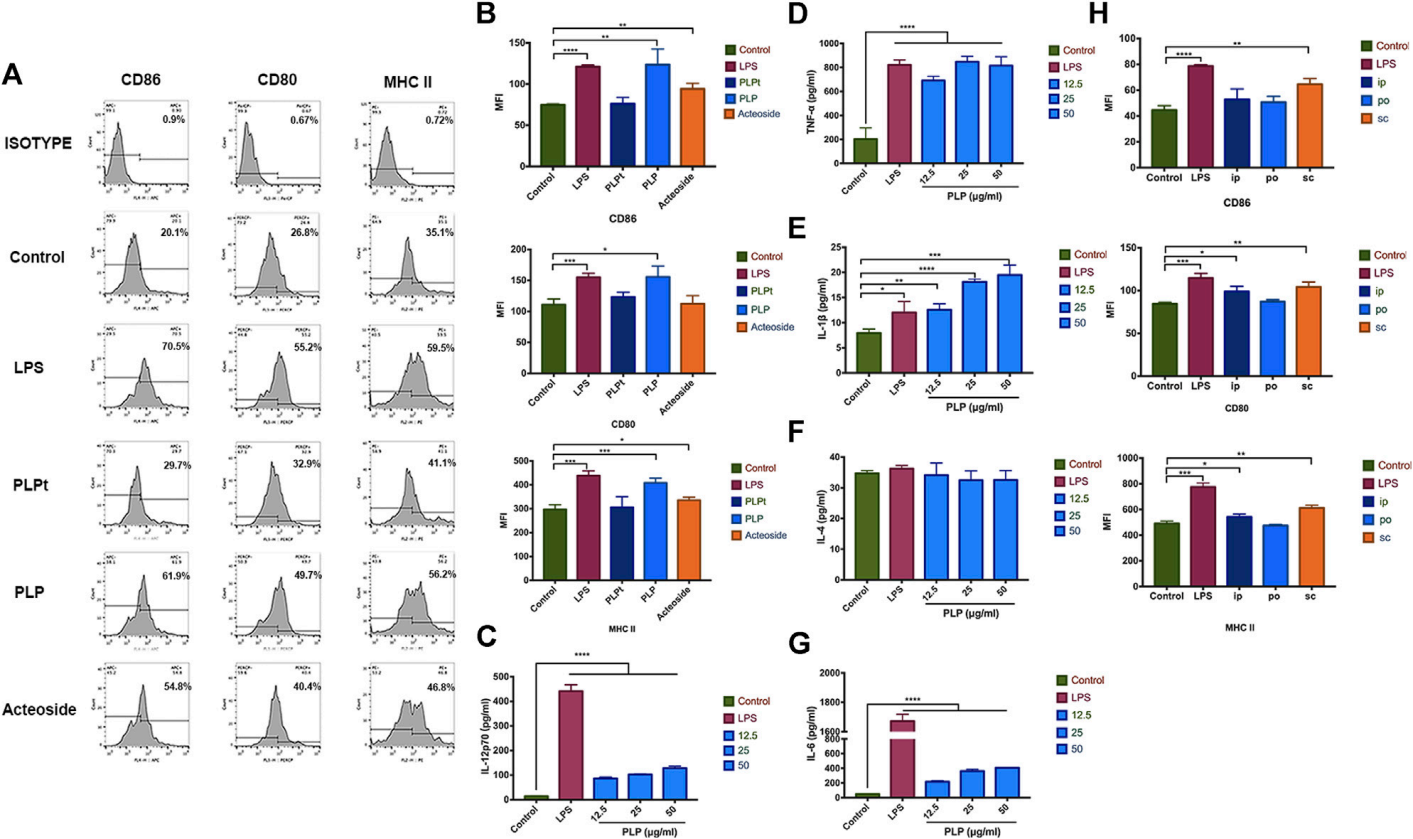
PLP-activated DCs stimulated the expression of costimulator. **(A)** BMDCs were analyzed for expression of CD86^+^, CD80^+^, and MHC II^+^ by flow cytometry after stimulated with 50 μg/ml PLP, PLPt, and acteoside for 48 h. **(B)** The mean fluorescence intensity (MFI) of three surface markers CD86, CD80, and MHC II expression, respectively, in different groups. Secretion of IL-12p70 **(C)**, TNF-α **(D)**, IL-1β **(E)**, IL-4 **(F)**, and IL-6 **(G)** from DCs treated with different formulations. **(H)** BMDCs were analyzed for the expression of CD86^+^, CD80^+^, and MHC II^+^ by flow cytometry after stimulated with 10 mg/kg PLP via s.c., i.p., and p.o. for 48 h. All values shown are the average values from three independent experiments ± SEM (*p < 0.05, **p < 0.01, and ***p < 0.001).

We determined whether PLP could induce DCs’ maturation *in vitro*. We studied the expression of DCs surface molecular markers CD86, CD80, and MHC II by flow cytometry. PLP, PLPt, and acteoside groups all revealed significantly upregulated expression of CD86, CD80, and MHC II, compared with the control group after 48 h treatment, and the PLP group was the most prominent among them (Figure 2A). Meanwhile, the mean fluorescence intensity (MFI) of three surface markers all increased after acteoside or PLP treatment. The MFI of CD86, CD80, and MHC II increased 1.66-, 1.40-, and 1.37-fold by PLP stimulation compared to the control group. As to acteoside, MFI of CD86 and MHC class II increased 1.26- and 1.13-fold, while no significant difference was observed in MFI of CD80 (Figure 2B). PLPt could slightly upregulate the expression percentage of MHC II and costimulatory molecules, but there was no significant difference between PLPt and medium control in MFI. These results indicated that, compared to PLPt and acteoside, PLP demonstrated a better maturation-promoting effect on DCs.

We next determined whether PLP could induce excretion of cytokines from DCs; DCs were stimulated with PLP or LPS for 48 h. The secretion of IL-12p70, TNF-α, IL-1β, and IL-6 was significantly enhanced by the PLP-treated DCs, compared to the control group (Figure 2C-E,G). PLP increased IL-12p70 secretion to 9.8-fold at 50 μg/ml. A 4-fold increase was observed for TNF-α in the PLP group. It was noted that PLP with 25 μg/ml and 50 μg/ml concentrations was as potent as LPS with 2 μg/ml concentration in the inductive efficacy of TNF-α’s secretion. Conversely, there was no significant difference in the secretion of IL-4 (Figure 2F), which indicated Th2 type immune response. It indicated that PLP could increase the secretion of Th1 cytokines such as IL-12p70, TNF-α, IL-1β, and IL-6 of DCs.

### PLP Induced DC Maturation *In Vivo*

Since PLP is effective in inducing the maturation of DCs *in vitro*, we further studied the administration routes to validate the safety and efficacy of PLP for DCs maturation *in vivo*. We administered PLP with 10 mg/kg dose via s.c., i.p., and p.o., as shown in Supplementary Figure S2, LPS (s.c) was used as a positive control. All groups of administration routes exhibited upregulated expression of CD86, CD80, and MHC II at Day 7, compared with the naive control group. S.c. route of PLP was the most effective route to activate DCs. When PLP was administered through i.p. or p.o. route, the efficacy was comparatively weaker than the s.c. administration route (Figure 2H). These results indicated that PLP was able to induce DCs maturation *in vivo*.

### PLP Strengthened DCs to Stimulate Lymphocyte Proliferation and CTL Differentiation

**FIGURE 3:**
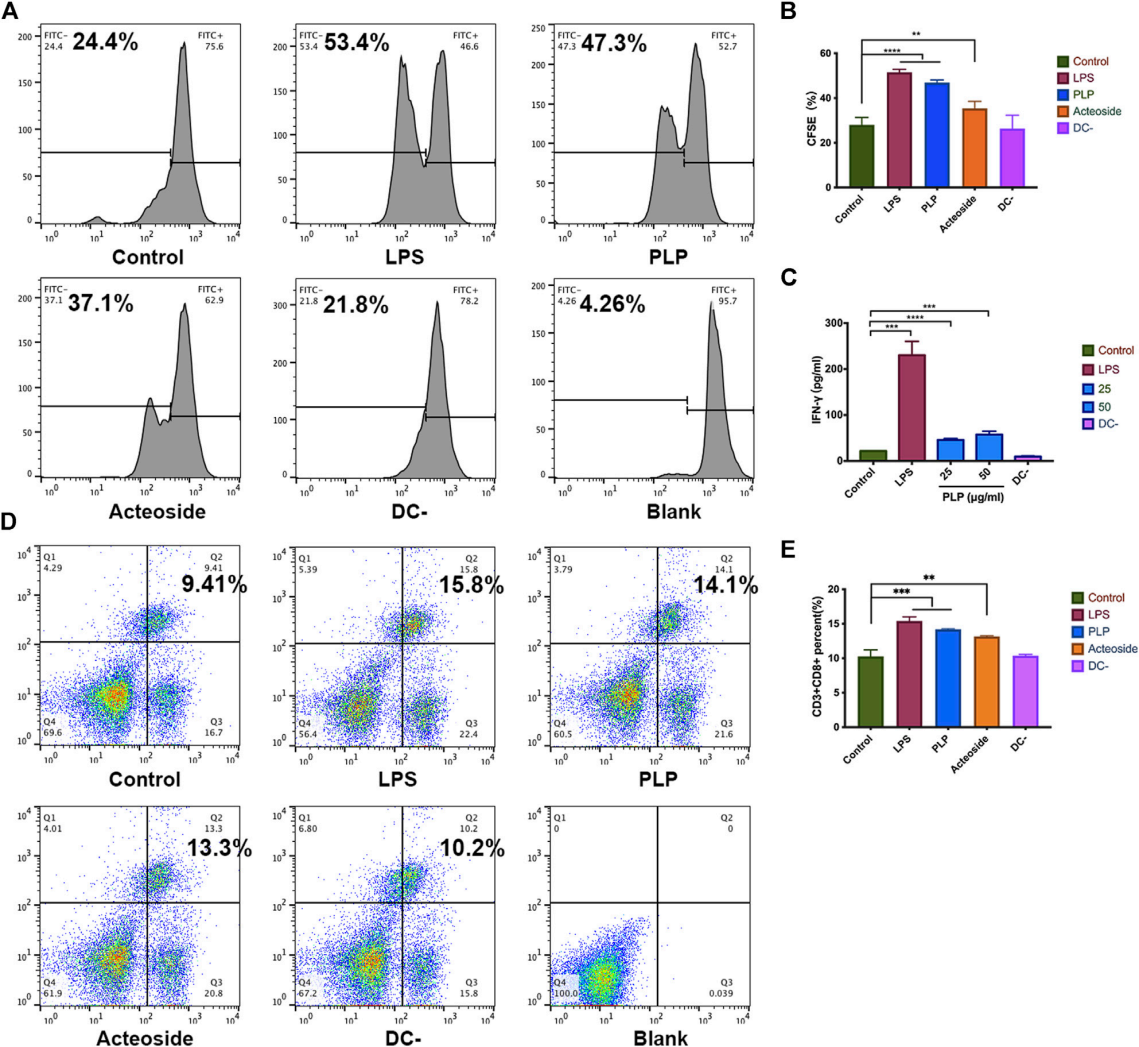
PLP strengthened DCs to stimulate lymphocyte proliferation and CTL differentiation. **(A, B)** CFSE-labeled spleen lymphocytes were stimulated with DCs for 48 h. The fraction of proliferative lymphocytes was measured in a CFSE dilution assay. Representative examples are shown. **(C)** The concentration of IFN-γ secreted by splenic lymphocytes. **(D, E)** Phenotype analysis of CD3^+^CD8^+^ T cells in the spleen. All values shown are the average values from three independent experiments ± SEM (*p < 0.05, **p < 0.01, and ***p < 0.001).

We studied the ability of PLP pretreated DCs on the stimulation of lymphocytes. We cocultured primed DCs using PLP with lymphocytes to determine if they can induce lymphocyte proliferation. The results showed that DCs activated by PLP and acteoside were relatively more potent in lymphocyte stimulation. It resulted in 47.3 and 37.1% increased rate of proliferation, compared to the control group of 24.4% (Figures 3A,B). The results suggested that PLP-treated DCs had the advantage to prime and activate lymphocytes.

We further studied the cytokine production of IFN-γ from coculture supernatants. More IFN-γ was detected in which lymphocytes were cocultured with PLP-stimulated DCs compared to unstimulated DCs after 48 h. IFN-γ produced by the spleen lymphocytes increased 4-fold at a PLP concentration of 50 μg/ml (Figure 3C). These results are consistent with that in the CFSE.

Moreover, we investigated whether PLP was attributable to CTL differentiation. Lymphocytes were incubated with anti-CD3 and anti-CD8 antibodies, and the percentage of CD3^+^CD8^+^ T lymphocytes was measured using flow cytometry. As shown in Figure 3D,E, an increased percentage of CD3^+^CD8^+^ T lymphocytes was observed in the PLP group (14.1%); though lower than that of the positive control (15.8%), it was still significantly higher than that of the medium group (9.41%). These results implied that PLP significantly elicited CD3^+^CD8^+^ T lymphocyte responses compared to medium controls.

### Growth Inhibitory Effects of PLP and Conditioned Medium on CIPp

**FIGURE 4:**
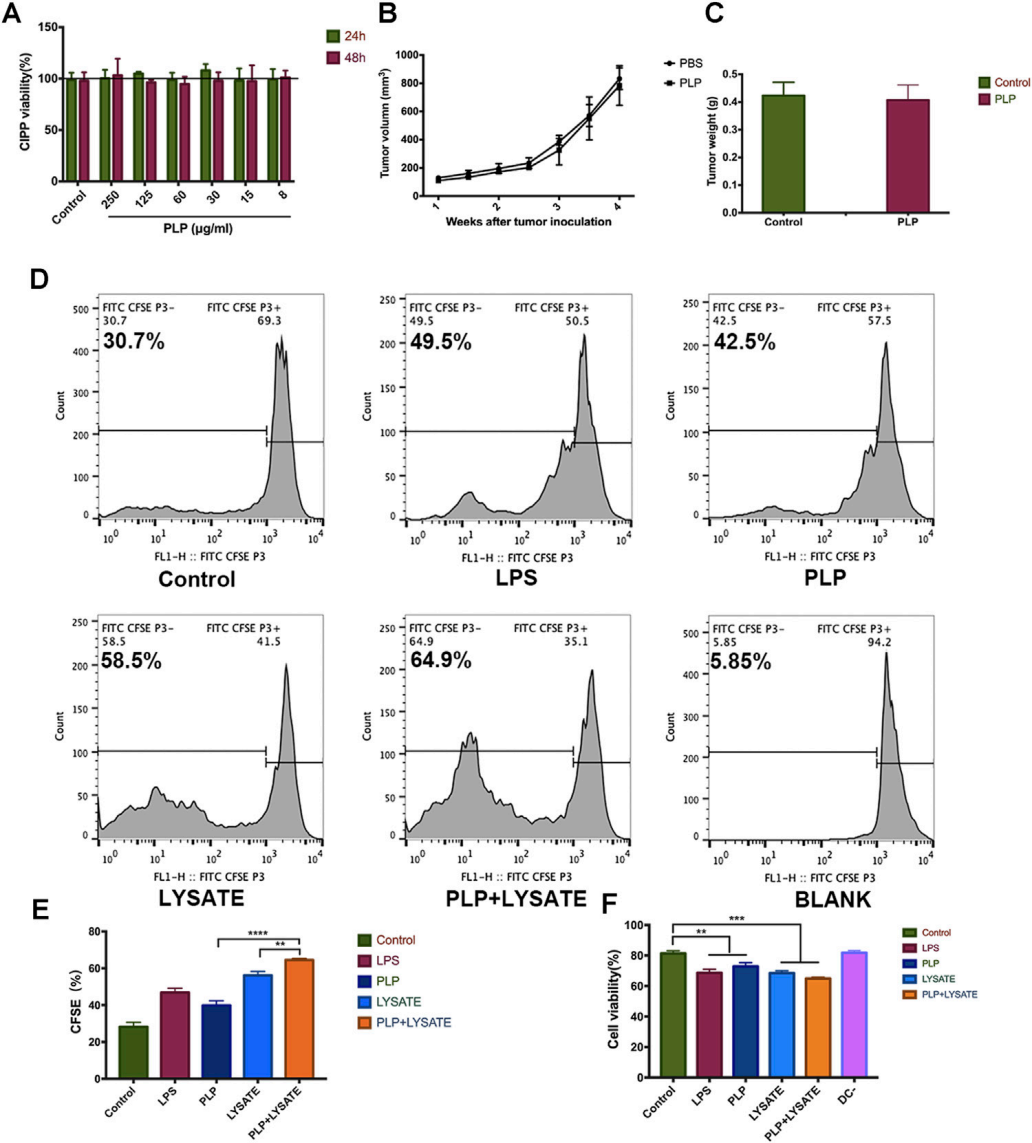
PLP enhanced tumor antigen-specific immune responses against tumor lysate-pulsed DCs. **(A)** Cytotoxic effects of CIPp cells were analyzed using Cell Counting Kit-8 at 8, 15, 30, 60, 125, and 250 μg/ml PLP treatment. **(B)** Mean tumor growth curves given by tumor volume in BALB/c nude mice. Mice were s.c. inoculated with CIPp cells (1 × 10^5^ cells). PLP (10 mg/kg/d, s.c.) was administered to the mice every 48 h, and the tumor growth was measured (n = 6). **(C)** The tumor weight was measured 28 days after tumor challenge in nude mice. **(D, E)** CFSE-labeled spleen lymphocytes were stimulated with DCs for 48 h. The fraction of proliferative lymphocytes was measured in a CFSE dilution assay. Representative examples are shown. **(F)** Cytotoxic effects of CIPp cells were analyzed using Cell Counting Kit-8 by different groups of conditioned medium (LPS, PLP, CIPp-cell-lysate, and CIPp-cell-lysate + PLP). All values shown are the average values from three independent experiments ± SEM (*p < 0.05, **p < 0.01, and ***p < 0.001).

We quantitatively evaluate the effects of PLP on the growth of CIPp cells using CCK-8 (Figure 4A). The results suggested that no obvious cytotoxicity on CIPp cells was observed after the treatment with different doses of PLP for 24 and 48 h. We did not find significant differences among all experimental groups.

To confirm the impact of PLP-associated T-cell immunity on the tumor inhibition and further verify the above results from the animal level, we administered PLP (10 mg/kg) to CIPp tumor-bearing BALB/c nude mice which reveal congenital defect of mature T cells. In this mice model, PLP did not significantly inhibit tumor growth 28 days after the administration (Figures 4B,C). These results suggest that T-cell immunity is necessary for the antitumor effect of subcutaneously administered PLP.

Next, we studied the influence of the PLP-induced antitumor activity on the interaction between DCs and lymphocytes using a CFSE dilution assay. The proliferation of spleen lymphocytes with PLP-induced DCs (42.5%) was higher than that of the control group (30.7%). And the proliferation of lymphocytes with CIPp-cell-lysate + PLP-pulsed DCs (64.9%) was higher than that of both the PLP group and CIPp-cell-lysate group (Figure 4D,E). It indicated that PLP + CIPp-cell-lysate may stimulate the proliferation of lymphocytes by promoting the maturation of DCs.

We studied the potential antitumor activity of the conditioned medium mediated by PLP on DCs, and CIPp cells were also subjected to different groups of conditioned medium for 48 h. The results of CCK-8 indicated that conditioned medium exerted growth inhibitory effects on CIPp cells, and the toxicity effect of the PLP group (27.1%) was much higher than that of the control group (18.6%). The results showed significant inhibitory effects of the CIPp-cell-lysate + PLP group, which had the most remarkable toxicity effect (35.1%), as compared to the PLP group and CIPp-cell-lysate group (Figure 4F). It suggested that the conditioned medium mediated by PLP had growth inhibitory effects on CIPp.

### PLP Achieved Therapeutic Effects and Improved Immunity in Breast Tumor Model

**FIGURE 5:**
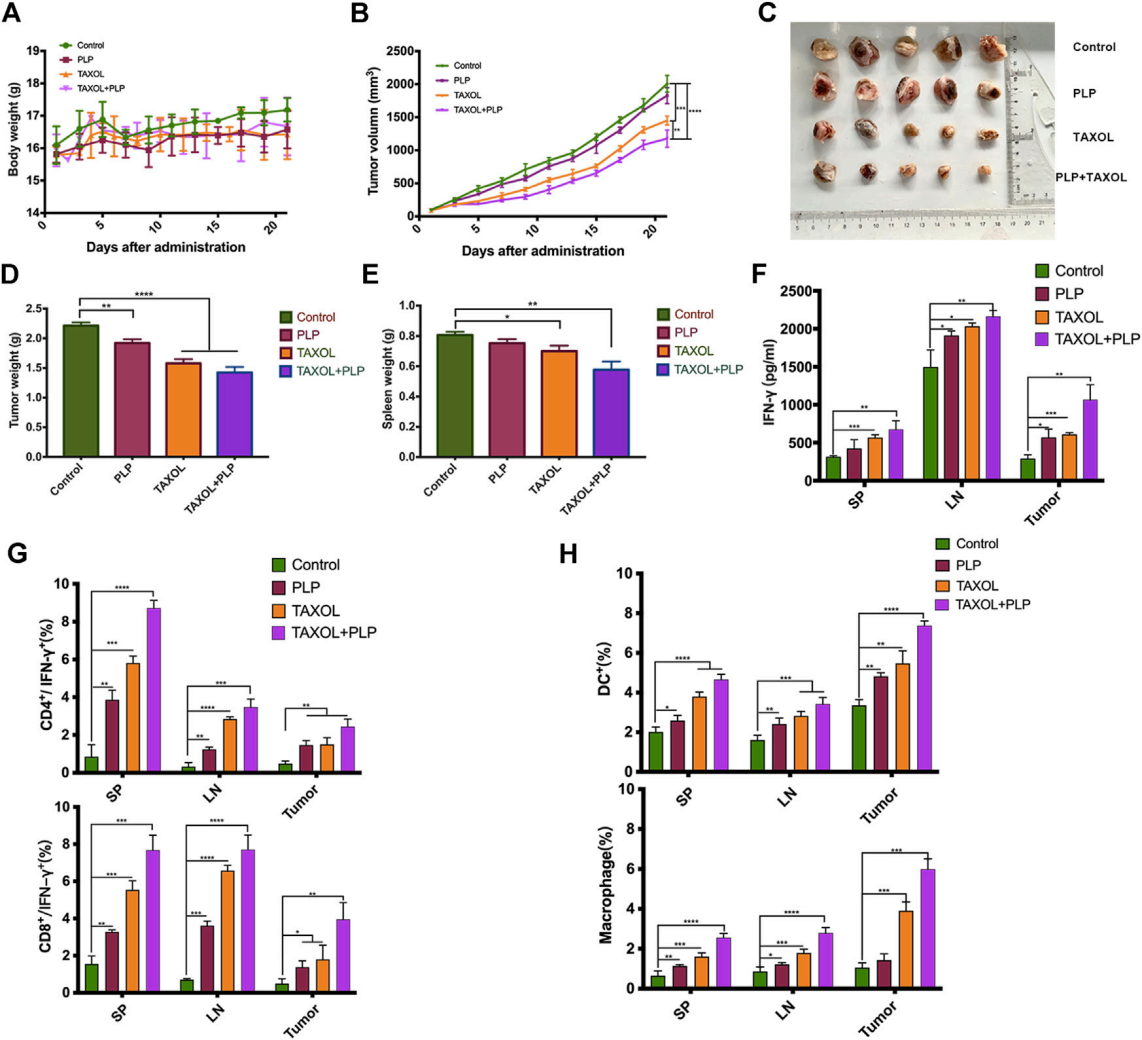
PLP achieved therapeutic effects and improved immunity in the breast tumor model. **(A)** Mice body weight, **(B)** mean tumor growth curves given by tumor volume, **(C)** images of excised tumors from each mouse in each treatment group, **(D)** tumor weight, and **(E)** the spleen weight during the total immunotherapy course. **(F)** IFN-γ released from cellular supernatant of tumor-bearing mice in control, PLP, TAXOL^®^, and TAXOL^®^+PLP groups was tested by ELISA. **(G)** Phenotype analysis of CD4^+^ IFN-γ^+^ T cells and CD8^+^ IFN-γ^+^ T cells and **(H)** DCs and macrophages in mice spleen, inguinal lymph node, and tumors 22 days after 4T1 tumor cell inoculation. All values shown are the average values from three independent experiments ± SEM (n = 5) (*p < 0.05, **p < 0.01, and ***p < 0.001).

To investigate the immunotherapy effects of PLP, the BALB/c mice were administered physiological saline, PLP, TAXOL^®^, and TAXOL^®^+PLP. There was no significant difference in mice body weight among all groups (Figure 5A). As shown in Figure 5B, TAXOL^®^+PLP significantly inhibited 4T1 tumor growth, compared with the control group and TAXOL group. Figure 5C showed the photos of tumor completely stripped from mice after sacrificed. Consistently, tumor weight and spleen weight in the TAXOL^®^+PLP group were significantly decreased at day 21 compared to the control group (Figures 5D,E). Expression of cellular supernatant IFN-γ was consistent with the antitumor results (Figure 5F).

To demonstrate that PLP induced antitumor immunity, we assessed whether IFN-γ-expressing CD4^+^ and CD8^+^ T cells in tumor-bearing mice increased due to administration of PLP on day 21 after tumor inoculation. Intracellular cytokine staining showed that mice administered TAXOL^®^+PLP had more IFN-γ-expressing CD4^+^ and CD8^+^ T cells in inguinal lymph node, spleens, and tumors as compared with the control group and TAXOL group. And the PLP group had more IFN-γ-expressing CD4^+^ T cells in inguinal lymph node and spleens and more IFN-γ-expressing CD8^+^ T cells in inguinal lymph node, spleen, and tumor as compared with the control group mice (Figure 5G; Supplementary Figures S3, 4). As shown in Figure 5H; Supplementary Figures S5, 6, the combination of PLP and TAXOL promoted more CD11c^+^ DCs and CD11b^+^F4/80^+^ macrophage aggregation in inguinal lymph node, spleen, and tumor as compared with the control group and TAXOL group. And the PLP group had more DCs and macrophages in inguinal lymph node, spleen, and tumor as compared with the control group. Representative gating scheme for flow cytometric analysis of immune cell populations was showed in Supplementary Figure S1. These results suggested that PLP achieved therapeutic effects and could improve immunity in the 4T1 breast tumor model.

## Discussion

Recent studies approved that polysaccharide components of Chinese herbal extracts exhibit the outstanding ability of immune modulation through activating DCs (Chen et al., 2006). In addition, besides polysaccharides, glycoside was also identified with the same activity. There were also reports which indicated that acteoside, another component isolated from *Plantago asiatica* L., could enhance immunity in mouse splenocytes (Kim et al., 2005). Thus, we first investigated if PLP and acteoside could activate the maturation of DCs. The results showed that PLPt, PLP, and acteoside all revealed significantly upregulated expression of MHC II and costimulatory molecules. However, at the same concentration, the PLP was the most prominent among them. It manifested that PLP had a stronger maturation-promoting effect on DCs than acteoside, which is consistent with reports by Huang et al. (2009).

In this study, we demonstrated that PLP performed the ability to upregulate DCs’ maturation marker and induce its functional maturation *in vivo* and *in vitro*. PLP enhanced the expression of surface molecules, including CD80, CD86, and MHC class II, suggesting that PLP induces the maturation of DCs. This finding was confirmed by the experiments *in vivo*. Groups administered with PLP via s.c., i.p., and p.o. all induced DCs’ maturation. Among the three routes of administration, PLP via s.c. route is the most effective, which is consistent with reports by Chen et al. (2009). Perhaps because the subcutaneously administered PLP was taken up by DCs present in hypodermis and lymphatics, which are transported to the sentinel lymph nodes and eventually enter the blood circulation and systemic lymphoid tissues more effectively. DCs primed by PLP cocultured with lymphocytes can further enhance the IL-12 p70 and TNF-α production. This indicates that PLP can induce cytotoxic T-cell activities *in vitro* and *in vivo* (Xiao et al., 2009; Carreno et al., 2013).

Admittedly, PLP is not as strong as LPS in terms of inducing IL-12 production, while it is comparable to LPS with the enhancement of lymphocyte proliferation. LPS is toxic and induces a severe inflammatory response. In contrast, PLP was prepared from the herb, and no substantial toxic effect was detected with relatively high doses in the cytotoxicity evaluation experiments (Figure 1C), which indicates a therapeutic safety for the doses pharmacologically. This advantage makes it perform the great potential to be a good supplement for immunosuppressed organisms. Improvement of immunity could be achieved by simply administrating PLP.

As the most important professional antigen-presenting cells, a crucial role of DCs is to present processed antigens to T cells so that T cells can develop a specific immune response to particular antigens (Martinez et al., 2007). We used spleen lymphocytes which were composed of T cells to better simulate the immune environment in the organism. The result demonstrated that DCs could significantly increase the proliferation of spleen lymphocytes when the ratio of DCs: lymphocytes was 1:4. We also used the ratio of 1:10, while the lymphocyte proliferation ability of DCs became weaker (data not shown). And this result is consistent with reports by Huang et al. (2009). The reason might be with the decrease of DCs, and there were not enough IL-2 secreted by DCs, resulting in less lymphocyte proliferation (Guermonprez et al., 2002). Besides, PLP-treated DCs induced higher levels of IFN-γ production from spleen lymphocytes *in vitro*, suggesting they were more potent in priming Th1 response. Immunogenic DCs can induce Th1 cell differentiation and/or CTL priming, depending on the constraints imposed by environmental modifiers, as well as the maturation signal they received (Reis e Sousa 2006).

The CCK-8 results showed that there was no obvious cytotoxicity on CIPp cells after the treatment with PLP (Figure 4A), which suggested that PLP could kill tumor cells via enhancing antitumor immunity rather than directly targeting cancer cells. In the BALB/c nude mice model which reveals congenital defect of mature T cells, PLP did not significantly inhibit tumor growth (Figure 4E), and it also suggested that T-cell immunity was necessary for the antitumor effect of PLP. We used PLP-activated DCs cocultured with spleen lymphocytes, and the conditioned medium was detected for potential antitumor activity. The results of CCK-8 showed that conditioned medium exerted growth inhibitory effects on CIPp cells. PLP and CIPp-cell-lysate jointly stimulated DCs cocultured with lymphocytes having the strongest killing effects on CIPp cells (Figure 4B). On the other hand, in the 4T1 tumor model, PLP and TAXOL^®^ group controlled the size of tumors and exerted better therapeutic effects than that administered TAXOL^®^ or PLP alone (Figure 5B), indicating that PLP can exert antitumor effects by upregulating immune responses and improving immunosuppression of the tumor microenvironment.

Antitumor immune responses can be initiated through both the innate and adaptive immune system. Innate immune cells, such as DCs, which process and present antigens to T lymphocytes, activate adaptive immunity. Macrophages can kill tumor cells indirectly by producing effector molecules and exerting phagocytosis (Wang et al., 2020). We chose TAXOL^®^ for combination because there was no general Chinese medicine immunotherapy drug as a positive control, and we also wanted to investigate whether PLP could enhance the effect of conventional chemotherapy medicine. The combination of PLP and TAXOL^®^ recruited more mature DCs and macrophages in inguinal lymph node, spleen, and tumor (Figure 5H). Mature DCs secret IL-12, which in turn acts on T cells and promotes Th1 cell differentiation. In addition, subcutaneously administered PLP not only induced IFN-γ expression in the lymph node but also in the spleen and tumor (Figure 5F). It suggested that PLP induced the tumor-specific T-cell response to a type 1 cytokine profile and results in successful cancer immunotherapy (Masuda et al., 2013). What is important is that effective anti-tumor immunity is generated during the infiltration of effector cells into tumor microenvironment, and antigen-specific T cells perform effector functions to kill tumor cells (Bos and Sherman 2010). Consistent with the enhancement of expression of IFN-γ, subcutaneously administered PLP significantly increased the infiltration of CD4^+^ IFN-γ^+^ and CD8^+^ IFN-γ^+^ T cells to the tumor sites (Figure 5G). These results suggested that PLP might recruit CD4^+^ and CD8^+^ T cells to the tumor sites to amplify the Th1 antitumor cellular immunity.

## Conclusion

Overall, PLP could promote the phenotypic and functional maturation of DCs both *in vivo* and *in vitro*. Mature DCs induced by PLP could enhance the cellular immune function, stimulate lymphocytes’ proliferation, and differentiate naive T cells into cytotoxic T cells, which had a cytotoxic effect on breast tumor cells. Tumor antigen-specific T-cell responses were enhanced by PLP and tumor lysate-pulsed DCs. In addition, PLP could achieve therapeutic effects and improve immunity in the breast tumor model. Our study provides scientific support and rationale on using PLP in various clinical conditions with poor immunity, especially for the tumor immune therapeutic effects in the future.

## Data Availability

The original contributions presented in the study are included in the article/Supplementary Material; further inquiries can be directed to the corresponding authors.
