# Supplementary material for: A Polysaccharide From the Whole Plant of Plantago asiatica L. Enhances the Antitumor Activity of Dendritic Cell-Based Immunotherapy Against Breast Cancer
Source: Front Pharmacol. 2021 Aug 24;12:678865. doi: 10.3389/fphar.2021.678865 (PMC8421731; doi:10.3389/fphar.2021.678865)
Supplement: Supplementary file 1 [file DataSheet1.docx]

Supplementary Material


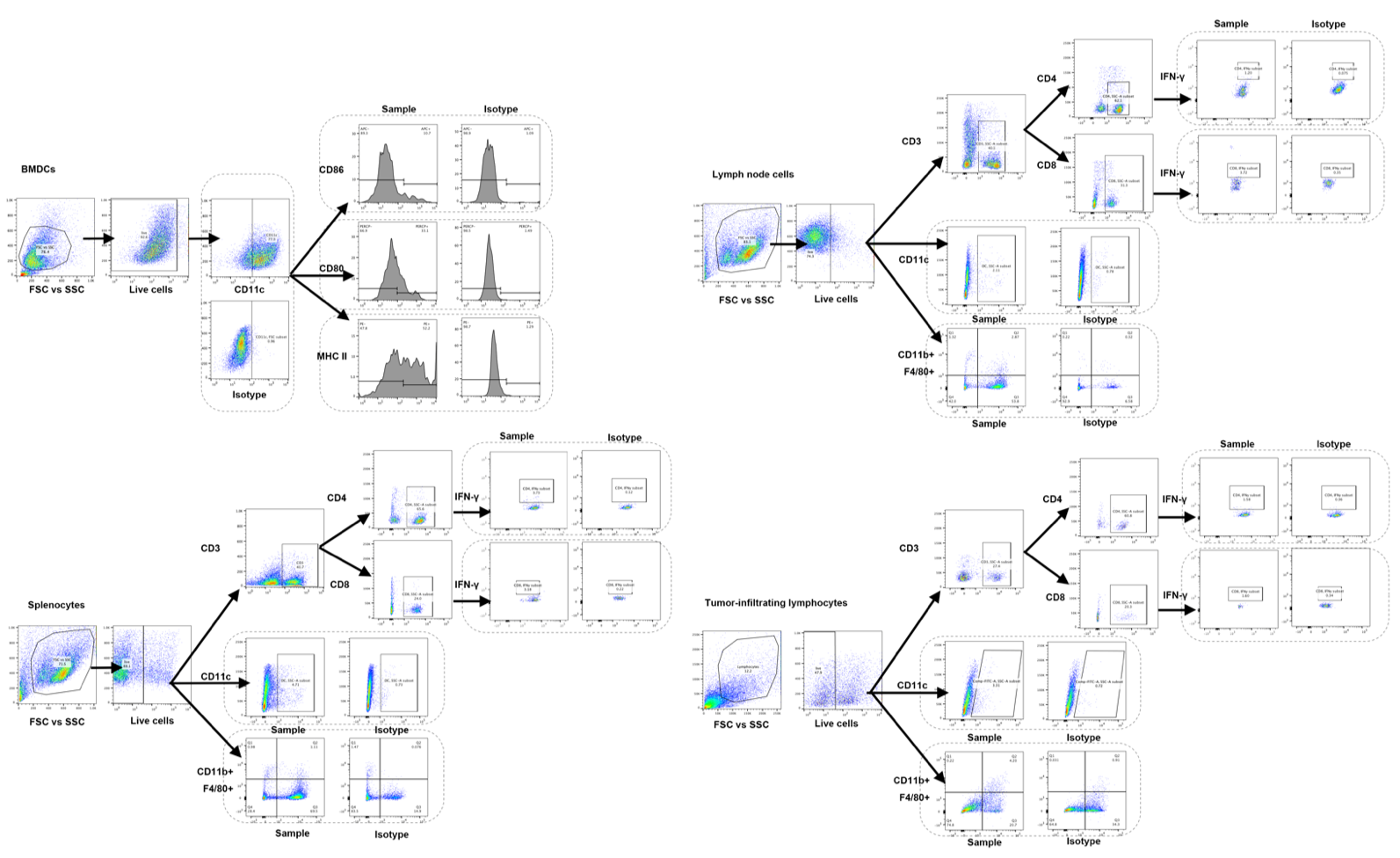


**Supplementary Figure 1.** Representative gating scheme for flow cytometric analysis of immune cell populations (bone marrow-derived DCs, splenocytes, lymph node cells, and tumor-infiltrating lymphocytes)


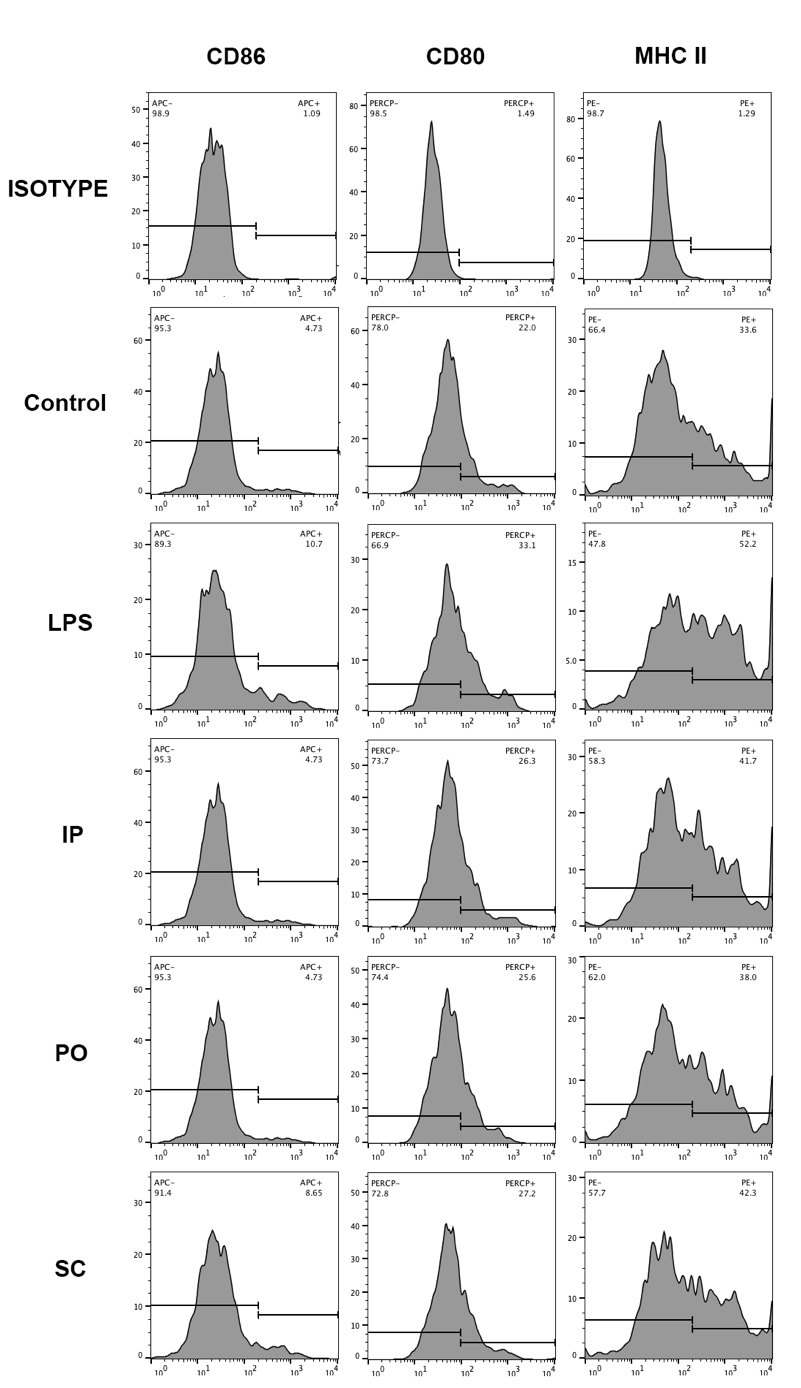


**Supplementary Figure 2.** The histogram profiles of CD86^+^, CD80^+^ and MHC II^+^ cells during different experimental groups, 48 h treatment with 10 mg/kg PLP via s.c., i.p. and p.o. all up-regulated expression of three surface markers.


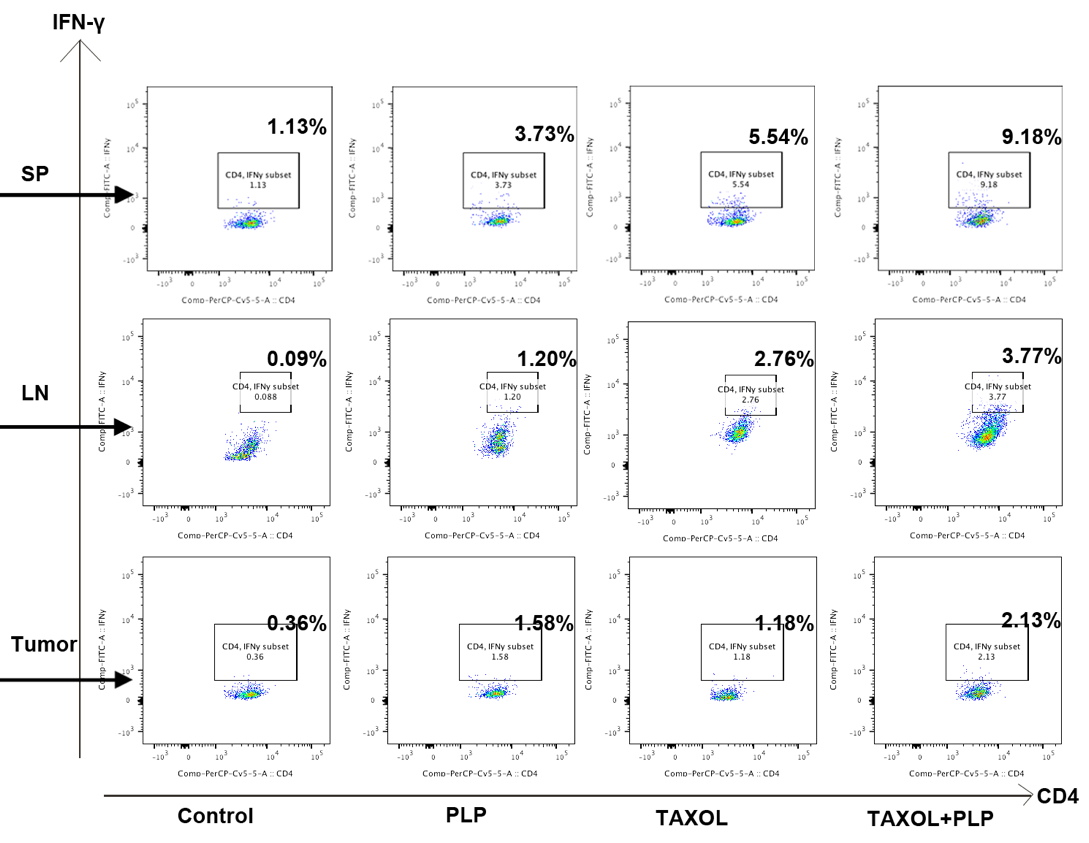


**Supplementary Figure 3.** Phenotype analysis of CD4^+^ IFN-γ^+^ T cells in mice spleen, inguinal lymph node and tumors 22 days after 4T1 tumor cells inoculation.

**
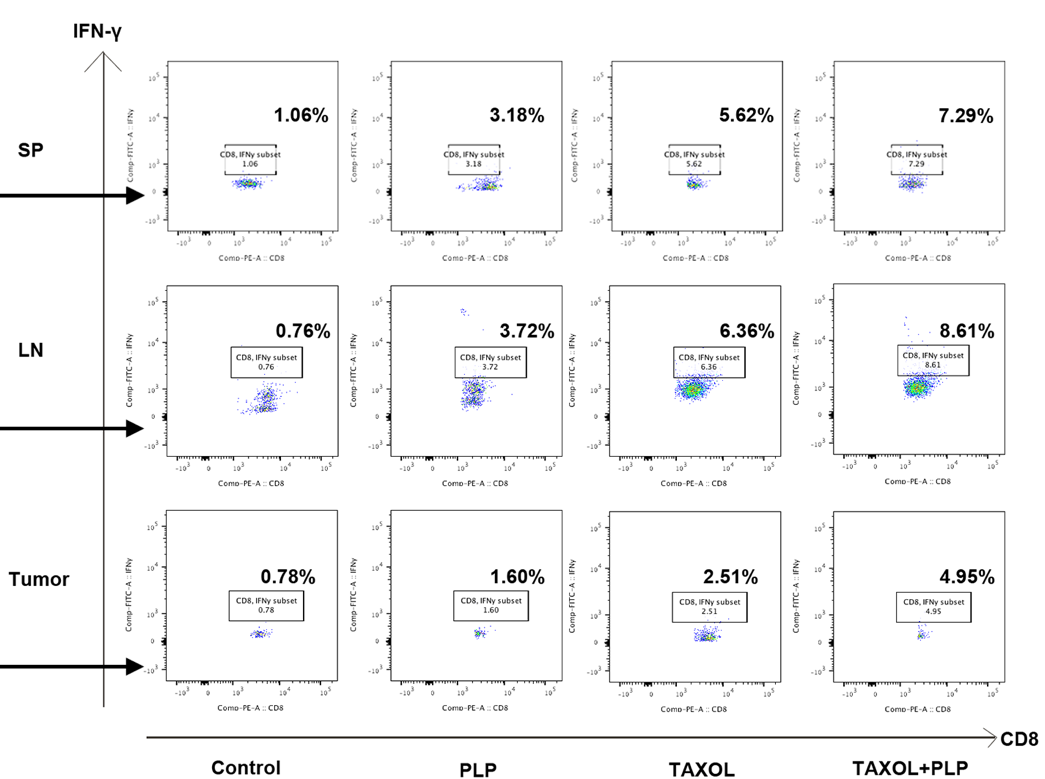
**

**Supplementary Figure 4.** Phenotype analysis of CD8^+^ IFN-γ^+^ T cells in mice spleen, inguinal lymph node and tumors 22 days after 4T1 tumor cells inoculation.

**
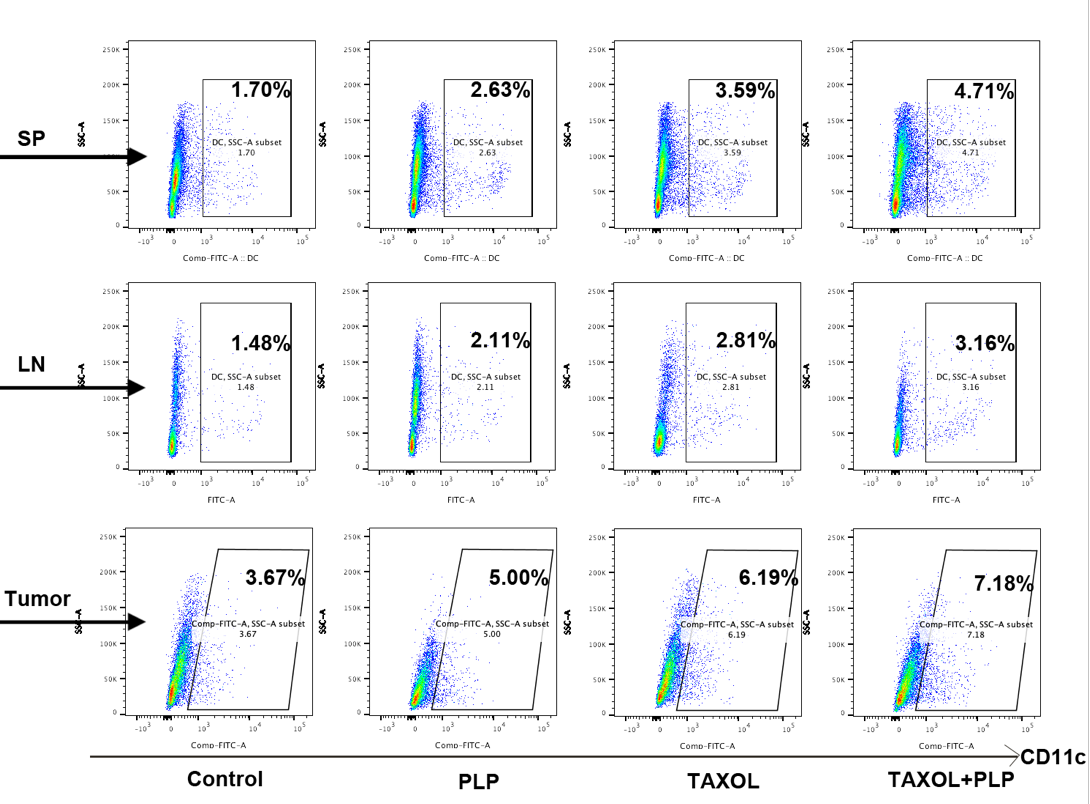
**

**Supplementary Figure 5.** DCs in mice spleen, inguinal lymph node and tumors 22 days after 4T1 tumor cells inoculation.

**
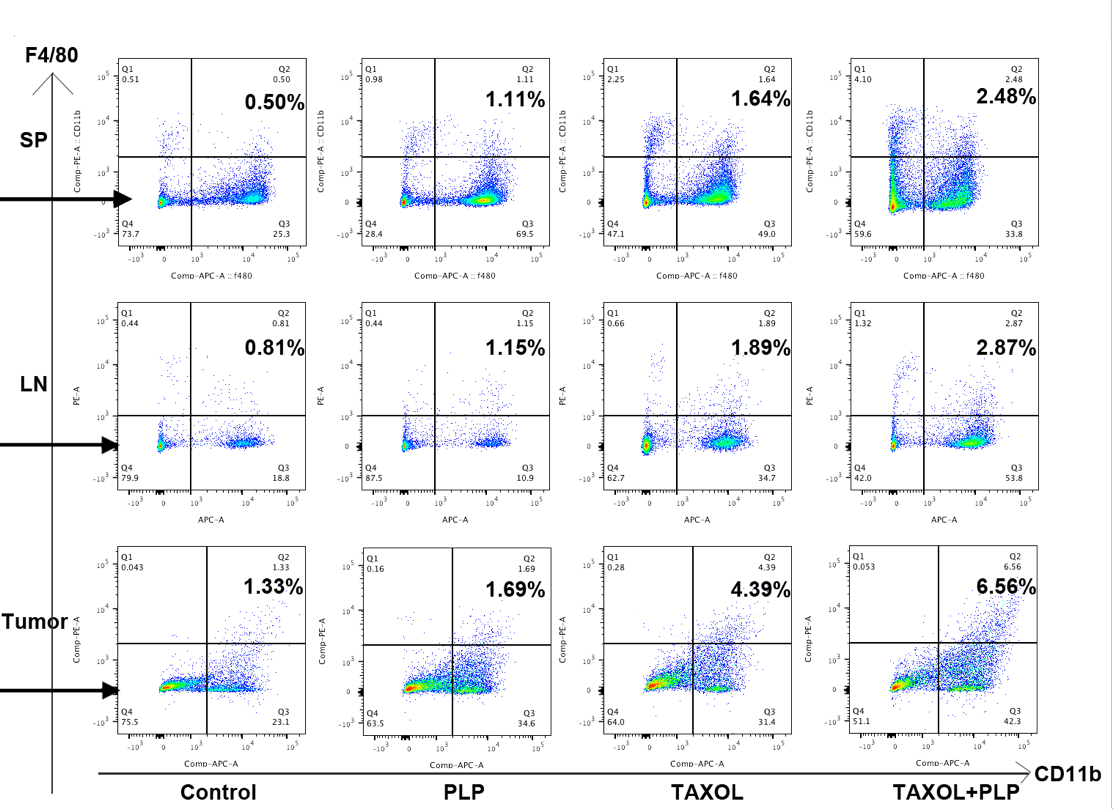
**

**Supplementary Figure 6.** Macrophages in mice spleen, inguinal lymph node and tumors 22 days after 4T1 tumor cells inoculation.
